# Impact of board-certified intensive care training facilities on choice of adjunctive therapies and prognosis of severe respiratory failure: a nationwide cohort study

**DOI:** 10.1186/s40560-024-00766-8

**Published:** 2024-12-19

**Authors:** Takuo Yoshida, Sayuri Shimizu, Kiyohide Fushimi, Takahiro Mihara

**Affiliations:** 1 Department of Health Data Science, Graduate School of Data Science, Yokohama City University, 22-2 Seto, Yokohama, Kanazawa 236-0027 Japan; 2 Department of Emergency Medicine, Jikei University School of Medicine, Minato-Ku, 105-8471 Japan; 3 Department of Health Policy and Informatics, Tokyo Medical and Dental University Graduate School, Tokyo, Japan; 4 Department of Anesthesiology, Yokohama City University School of Medicine, Yokohama, 236-0004 Japan

**Keywords:** Acute respiratory distress syndrome, Clinical practice, Intensivist, National in-patient database, Severe respiratory failure

## Abstract

**Background:**

Patients with severe respiratory failure have high mortality and need various interventions. However, the impact of intensivists on treatment choices, patient outcomes, and optimal intensivist staffing patterns is unknown. In this study, we aimed to evaluate treatments and clinical outcomes for patients at board-certified intensive care training facilities compared with those at non-certified facilities.

**Methods:**

This retrospective cohort study used Japan’s nationwide in-patient database from 2016 to 2019 and included patients with non-operative severe respiratory failure who required mechanical ventilation for over 4 days. Treatments and in-hospital mortality were compared between board-certified intensive care facilities requiring at least one intensivist and non-certified facilities using propensity score matching.

**Results:**

Of the 66,905 patients in this study, 30,588 were treated at board-certified facilities, and 36,317 were not. The following differed between board-certified and non-certified facilities: propofol (35% vs. 18%), dexmedetomidine (37% vs. 19%), fentanyl (50% vs. 20%), rocuronium (8.5% vs. 2.6%), vecuronium (1.9% vs. 0.6%), noradrenaline (35% vs. 19%), arginine vasopressin (8.1% vs. 2.0%), adrenaline (2.3% vs. 1.0%), dobutamine (8.7% vs. 4.8%), phosphodiesterase inhibitors (1.0% vs. 0.3%), early enteral nutrition (29% vs. 14%), early rehabilitation (34% vs. 30%), renal replace therapy (15% vs. 6.7%), extracorporeal membrane oxygenation (1.6% vs. 0.3%), critical care unit admission (74% vs. 30%), dopamine (9.0% vs. 15%), sivelestat (4.1% vs. 7.0%), and high-dose methylprednisolone (13% vs. 15%). After 1:1 propensity score matching, the board-certified group had lower in-hospital mortality than the non-certified group (31% vs. 38%; odds ratio, 0.75; 95% confidence interval, 0.72–0.77; *P* < 0.001). Subgroup analyses showed greater benefits in the board-certified group for older patients, those who required vasopressors on the first day of mechanical ventilation, and those treated in critical care units.

**Conclusions:**

Board-certified intensive care training facilities implemented several different adjunctive treatments for severe respiratory failure compared to non-board-certified facilities, and board-certified facilities were associated with lower in-hospital mortality. Because various factors may contribute to the outcome, the causal relationship remains uncertain. Further research is warranted to determine how best to strengthen patient outcomes in the critical care system through the certification of intensive care training facilities.

**Supplementary Information:**

The online version contains supplementary material available at 10.1186/s40560-024-00766-8.

## Background

Patients with severe respiratory failure have a high mortality of ~ 40% and require diverse interventions such as adjunct therapies, prone positioning, nutrition, mobilization, medical devices, and medications [1–4]. Due to numerous treatment options for severe respiratory failure, significant variations in clinical practice have been reported, including implementation rates for lung-protective ventilation and the adoption of adjunctive therapies across hospitals and regions [5, 6]. Among those variations, intensivists are expected to make evidence-based decisions to improve patient outcomes.

Studies on intensivist staffing patterns in intensive care units (ICU) have shown conflicting results on their impact on patient prognosis [7–14]. High-intensity intensivist staffing, featuring either an intensivist-led team or mandatory intensivist consultation, has been reported to be associated with lower mortality than low-intensity intensivist staffing [11–13, 15]. However, extended intensivist coverage time did not consistently improve patient outcomes; some studies showed no significant mortality reduction with 24-h staffing compared with that with daytime-only coverage [7–10, 16, 17]. Furthermore, how different intensivist staffing patterns produce treatment differences remains unclear. Differences in the treatment of severe respiratory failure between intensivists and non-intensivists and the impact of these differences on patient outcomes have not been studied thoroughly yet. Examining the outcomes along with treatments for severe respiratory failure, which has numerous treatment options but a poor prognosis, can help understand the impact of intensivists on patient outcomes.

To address these uncertainties, we conducted a retrospective cohort study using a nationwide administrative in-patient database. This study aimed to determine differences in the treatment and prognosis of severe respiratory failure between board-certified intensive care training facilities that required at least one intensivist in an ICU and non-board-certified facilities.

## Methods

We conducted a retrospective cohort study using a nationwide administrative in-patient database in Japan approved by the Ethics Committee of Yokohama City University (F220300054; approved July 22, 2022). This study adhered to the ethical standards of the responsible committee on human experimentation (institutional or regional) and the 1975 Helsinki Declaration. Informed consent from individual patients was waived because of the anonymous nature of the data, and the study was conducted in accordance with the Strengthening the Reporting of Observational Studies in Epidemiology statement [18].

### Data source

We obtained relevant data from the Japanese Diagnosis Procedure Combination (DPC) in-patient database, which came to include discharge abstracts and administrative claims data for ~ 1200 acute-care hospitals as of 2019, covering ~ 90% of all tertiary-care emergency hospitals in Japan [19]. At hospital discharge, the attending physician recorded the following clinically relevant data that the database captured for each patient: age, sex, diagnoses, daily procedures (Japanese medical procedure codes), drugs administered daily, and admission and discharge statuses. The database categorizes six diagnostic groups, each limited to specific recordable diseases, using the International Classification of Diseases, Tenth Revision codes (ICD-10). Diagnoses are coded under the four classifications “main diagnosis”, “admission-precipitating diagnosis”, “most resource-consuming diagnosis”, and “second resource-consuming diagnosis”. Additionally, up to four diagnoses may be coded as “comorbidities present at admission” or “conditions arising after admission”. It is essential to assess whether the disease names recorded in the DPC database correspond with clinical diagnoses. Various validation studies of the DPC database have shown high sensitivity and specificity for documenting procedures and high specificity with moderate sensitivity for diagnoses [20, 21]. The sensitivity for the diagnosis of respiratory diseases was 22–100%, whereas specificity consistently exceeded 95% [22].

### Study population

Patients discharged between April 1, 2016, and March 31, 2020, were screened for inclusion. The inclusion criteria included (i) mechanical ventilation (MV) for 4 consecutive days within the first 7 days of admission and (ii) a diagnosis of acute respiratory diseases with possible bilateral lesions (recorded in “main diagnosis”, “admission-precipitating diagnosis”, “most resource-consuming diagnosis”, “second resource-consuming diagnosis”, or “comorbidities present at the time of admission”, see Table S1 for a list of diagnostic codes). A 4-day duration of MV was used as a surrogate for severe hypoxemia due to the DPC data limitations that offered no details beyond 4 days and were supported by previous acute respiratory distress syndrome research [1]. Disease names indicating possible bilateral lesions were clinically determined and manually extracted from the ICD-10 codes. In clinical practice, it is often challenging to diagnose a single definitive cause of severe respiratory failure, and the past ARDS diagnostic criteria did not require the identification of an underlying disease aside from congestive heart failure. Based on these considerations, all types of acute respiratory failure diseases were included in our study. The exclusion criteria included (i) age < 18 years, (ii) cardiopulmonary resuscitation on the first day of MV, (iii) diagnosis of heart failure or congestion (recorded in “main diagnosis”, “admission-precipitating diagnosis”, “most resource-consuming diagnosis”, or “second resource-consuming diagnosis”, see Table S1 for a list of diagnostic codes), and (iv) any surgery performed other than tracheostomy. Postsurgical cases were excluded due to the DPC data's lack of specific reasons for MV, addressing potential non-respiratory failure-related MV needs.

### Exposure variables

Patients treated at a hospital with a board-certified intensive care training facility were included in the certified group. Patients in the certified group were not necessarily treated at the ICU, even if they were admitted to that hospital. Accreditation of board-certified intensive care training facilities by the Japanese Society of Intensive Care Medicine (JSICM) during the study period required at least one full-time intensivist in an ICU with a minimum of four beds, regardless of the treatment modalities provided. Board certification status was determined by referencing the annual report in JSICM’s domestic journal [23].

### Covariates

We collected data, including age, sex, body mass index, emergency admission, ambulance use, smoking status, diagnosis of acute respiratory disease, and 17 diagnoses for the Charlson Comorbidity Index [24]. We also collected the use of vasopressors and extracorporeal membrane oxygenation on the first day of MV to assess the severity of respiratory and circulatory failure.

### Treatment after mechanical ventilation initiation

We collected data on treatments administered within the first week of MV, including sedatives (dexmedetomidine, propofol, midazolam), opioids (fentanyl, morphine), neuromuscular blockers (rocuronium, vecuronium), vasopressors (noradrenaline, dopamine, arginine vasopressin), inotropic agents (dobutamine, phosphodiesterase inhibitors, adrenaline), sivelestat (approved in Japan for acute lung injury), methylprednisolone categorized by dosage (high-dose ≥ 500 mg, low-dose ≤ 200 mg), antibiotics, and antifungal drugs. For sedative drugs, opioids, neuromuscular blockers, and vasopressors, the collected information on the first day of MV was excluded to avoid confusion with medications used for intubation. Additional data collected included bronchoalveolar lavage during hospitalization, early enteral nutrition (defined as implementation within 48 h of MV initiation), enteral nutrition within 7 days, parenteral nutrition within 7 days, early rehabilitation (defined as implementation within 72 h of MV initiation), extracorporeal membrane oxygenation during hospitalization, renal replacement therapy during hospitalization, ICU admission, and high-care unit (HCU) admission. The ICU was defined as a separate unit for critically ill patients staffed with at least one onsite physician around the clock and a nurse-to-patient ratio of 1:2. The HCU is a separate unit with at least one in-hospital physician available 24/7 and a nurse-to-patient ratio of 1:4 or 1:5. A critical care unit admission was defined as any case in which a patient was admitted in either the ICU or HCU.

### Outcome variables

The primary outcome was in-hospital mortality, and secondary outcomes included reintubation within 7 days of extubation, tracheostomy during hospitalization, total MV duration, ICU stay length, HCU stay length, and overall hospital stay. Additionally, for survivors, MV dependency was assessed at discharge, which was defined as ongoing invasive MV.

### Statistical analysis

The study results are presented as median (interquartile range [IQR]) or number (%), as appropriate. Missing data for four baseline characteristics—weight in 4856 cases (7.3%), height in 6215 cases (9.3%), ambulance use in 71 cases (0.1%), and smoking status in 10,510 cases (16%)—were assumed to be missing at random and were imputed by the missForest algorithm [25, 26], using all variables except timing of tracheostomy and CCU length of stay listed in Tables 1, 2, 3, and sTable 2 as predictors. This method, which is based on a random forest, iteratively handles missing data [25, 26]. It starts with the column with the fewest missing values, filling other columns with mean values to use as predictors. This process is repeated for each column until a stopping criterion is met. In our study, a single dataset created using this method enabled the main analysis, sensitivity analysis, and subgroup analysis to be conducted. We performed propensity score matching to adjust for confounding by indication and the potential baseline differences between the certified and non-certified groups [27, 28]. The propensity score for treatment at a hospital with a board-certified intensive care training facility was calculated using the logistic regression model confounders listed in Table 1 and Table S2 (age, sex, body mass index, emergency admission, ambulance use, smoking status, diagnosis of acute respiratory disease, 17 diagnoses for the Charlson Comorbidity Index, and the use of vasopressors on the first day of MV, and extracorporeal membrane oxygenation on the first day of MV). Propensity score matching was used to balance the two groups in a 1:1 ratio without replacement. This was achieved using the nearest neighbor algorithm with the caliper width set to 0.10 of the standard deviation of the logit of the propensity scores. Group balance was assessed using the absolute standardized difference. Covariates with absolute standardized differences < 0.10 were considered well-balanced. All reported P-values were two-sided, with a P-value of less than 0.05 considered statistically significant. Owing to the potential for type I errors from multiple comparisons, findings from secondary outcomes and subgroup analyses were considered exploratory. Missing data imputation was performed using R (version 4.1.0; R Foundation for Statistical Computing, Vienna, Austria), and all other statistical analyses were performed using STATA version 16.1 (StataCorp, College Station, TX, USA).

**Table 1:** Baseline characteristics of patients with severe respiratory failure on mechanical ventilation

	Unmatched groups			Propensity score-matched groups		
	Certified (*n* = 30,588)	Non-certified (*n* = 36,317)	ASD	Certified (*n* = 26,673)	Non-certified (*n* = 26,673)	ASD
Age, years	73 (62–81)	77 (69–84)	0.326	75 (65–82)	75 (66–83)	0.046
Male sex, *n* (%)	19,745 (65)	22,982 (63)	0.026	17,170 (64)	17,221 (65)	0.004
Body mass index, kg/m^2^	21 (18–24)	20 (18–23)	0.125	20 (18–24)	21 (18–24)	0.029
Emergency admission, *n* (%)	29,154 (95)	34,215 (94)	0.049	25,332 (95)	25,298 (95)	0.006
Ambulance use, *n* (%)	22,555 (74)	22,645 (62)	0.248	19,029 (72)	18,921 (71)	0.010
Smoking, *n* (%)	9357 (38)	11,603 (37)	0.018	8145 (37)	8708 (38)	0.006
Diagnosis associated with acute respiratory disease^a^, *n* (%)						
Bacterial pneumonia	11,869 (39)	12,730 (35)	0.078	9984 (37)	9815 (37)	0.013
Interstitial pneumonia	4038 (13)	4934 (14)	0.011	3649 (14)	3753 (14)	0.011
ARDS	2492 (8.1)	2778 (7.6)	0.018	2161 (8.1)	2172 (8.1)	0.002
Allergic	1837 (6.0)	3082 (8.5)	0.096	1743 (6.5)	1785 (6.7)	0.006
Viral pneumonia	2020 (6.6)	2012 (5.5)	0.045	1694 (6.4)	1635 (6.1)	0.009
Tuberculosis	549 (1.8)	1003 (2.8)	0.065	522 (2.0)	532 (2.0)	0.003
*Mycobacterium avium* complex	269 (0.9)	515 (1.4)	0.051	263 (1.0)	261 (1.0)	0.001
Aspergillus	342 (1.1)	358 (1.0)	0.013	286 (1.1)	281 (1.1)	0.002
Pneumocystis pneumonia	323 (1.1)	229 (0.6)	0.047	248 (0.9)	215 (0.8)	0.013
Any pneumonia or bronchitis^b^	21,790 (71)	26,374 (73)	0.031	19,169 (72)	19,238 (72)	0.006
Vasopressors on MV Day 1,* n* (%)	11,037 (36)	8787 (24)	0.261	8631 (32)	8187 (31)	0.036
ECMO on MV Day 1,* n* (%)	21 (0.1)	0 (0)	0.037	14 (0.1)	0 (0)	0.032

*ARDS* acute respiratory distress syndrome, *MV* mechanical ventilation, *ECMO* extracorporeal membrane oxygenation, *ASD* absolute standardized difference

^a^Diagnosis recorded in “main diagnosis”, “admission-precipitating diagnosis”, “most resource-consuming diagnosis”, or “second resource-consuming diagnosis”. See Table S1 for the list of diagnostic codes. Some patients were assigned multiple diagnoses in several categories

^b^Any pneumonia or bronchitis without specified causative microorganisms

**Table 2:** Interventions for patients with severe respiratory failure on mechanical ventilation

	Unmatched groups			Propensity score-matched groups		
	Certified (n = 30,588)	Non-certified (n = 36,317)	*P* value	Certified (*n* = 26,673)	Non-certified (*n* = 26,673)	*P* value
Sedative, *n* (%)	18,727 (61)	15,349 (42)	< 0.001	15,743 (59)	12,633 (47)	< 0.001
Propofol	10,711 (35)	6436 (18)	< 0.001	8831 (33)	5545 (21)	< 0.001
Dexmedetomidine	11,291 (37)	6842 (19)	< 0.001	9479 (36)	5617 (21)	< 0.001
Midazolam	8143 (27)	7847 (22)	< 0.001	6658 (25)	6516 (24)	0.150
Opioid, *n* (%)	15,863 (52)	7934 (22)	< 0.001	13,189 (49)	6854 (26)	< 0.001
Fentanyl	15,400 (50)	7175 (20)	< 0.001	12,750 (48)	6318 (24)	< 0.001
Morphine	632 (2.1)	817 (2.2)	0.10	578 (2.2)	584 (2.2)	0.86
Neuromuscular blockade, *n* (%)	3132 (10)	1182 (3.3)	< 0.001	2546 (9.5)	1055 (4.0)	< 0.001
Rocuronium	2602 (8.5)	962 (2.6)	< 0.001	2130 (8.0)	860 (3.2)	< 0.001
Vecuronium	578 (1.9)	225 (0.6)	< 0.001	452 (1.7)	200 (0.7)	< 0.001
Vasopressor, *n* (%)	12,471 (41)	10,731 (30)	< 0.001	10,172 (38)	9370 (35)	< 0.001
Noradrenaline	10,734 (35)	6701 (19)	< 0.001	8673 (33)	6063 (23)	< 0.001
Arginine vasopressin	2473 (8.1)	710 (2.0)	< 0.001	1922 (7.2)	677 (2.5)	< 0.001
Dopamine	2751 (9.0)	5469 (15)	< 0.001	2309 (8.7)	4623 (17)	< 0.001
Adrenaline	698 (2.3)	351 (1.0)	< 0.001	550 (2.1)	312 (1.2)	< 0.001
Inotropic agent, *n* (%)	2834 (9.3)	1795 (4.9)	< 0.001	2380 (8.9)	1471 (5.5)	< 0.001
Dobutamine	2650 (8.7)	1729 (4.8)	< 0.001	2232 (8.4)	1429 (5.4)	< 0.001
PDE inhibitor	313 (1.0)	118 (0.3)	< 0.001	253 (0.9)	81 (0.3)	< 0.001
Sivelestat, *n* (%)	1255 (4.1)	2548 (7.0)	< 0.001	1096 (4.1)	2057 (7.7)	< 0.001
High-dose methylprednisolone, *n* (%)	4074 (13)	5415 (15)	< 0.001	3587 (13)	4259 (16)	< 0.001
Low-dose methylprednisolone, *n* (%)	5359 (18)	6582 (18)	0.042	4756 (18)	4805 (18)	0.580
Antibiotics^a^, *n* (%)	28,153 (92)	32,354 (89)	< 0.001	24,414 (92)	24,079 (90)	< 0.001
Antifungal drugs^b^, *n* (%)	1373 (4.5)	1011 (2.8)	< 0.001	1130 (4.2)	843 (3.2)	< 0.001
BAL during hospitalization, *n* (%)	726 (2.4)	484 (1.3)	< 0.001	604 (2.3)	404 (1.5)	< 0.001
Early EN^c^, *n* (%)	8758 (29)	5092 (14)	< 0.001	7396 (28)	4271 (16)	< 0.001
EN within 7 days, *n* (%)	16,361 (54)	10,178 (28)	< 0.001	13,824 (52)	8518 (32)	< 0.001
PN within 7 days, *n* (%)	5226 (17)	6440 (18)	0.028	4374 (16)	5211 (20)	< 0.001
Early rehabilitation^d^, *n* (%)	10,289 (34)	10,860 (30)	< 0.001	9021 (34)	7749 (29)	< 0.001
ECMO during hospitalization, *n* (%)	503 (1.6)	101 (0.3)	< 0.001	369 (1.4)	96 (0.4)	< 0.001
RRT during hospitalization, *n* (%)	4563 (15)	2445 (6.7)	< 0.001	3634 (14)	2144 (8.0)	< 0.001
Tracheostomy, *n* (%)	7753 (25)	7445 (21)	< 0.001	6700 (25)	5665 (21)	< 0.001
Timing of tracheostomy, day	11 (8.0–6)	14 (9.0–19)	< 0.001	11 (8.0–16)	13 (9.0–19)	< 0.001
Critical care units use, *n* (%)	22,627 (74)	10,881 (30)	< 0.001	19,241 (72)	9157 (34)	< 0.001
Intensive care unit use	17,513 (57)	7826 (22)	< 0.001	14,663 (55)	6580 (25)	< 0.001
High-care unit use	8844 (29)	3487 (9.6)	< 0.001	7676 (29)	2954 (11)	< 0.001

Data are presented as n (%) or median (interquartile range)

*PDE* phosphodiesterase, *BAL* bronchoalveolar lavage, *EN* enteral nutrition, *PN* parenteral nutrition, *ECMO* extracorporeal membrane oxygenation, *RRT* renal replacement therapy

^a^Penicillins, cephalosporins, carbapenems, anti-*methicillin-resistant Staphylococcus aureus* drugs, quinolones, aminoglycosides, and others

^b^Micafungin and caspofungin

^c^Implementation within 48 h of mechanical ventilation initiation

^d^Implementation within 72 h of mechanical ventilation initiation

**Table 3:** Outcomes of patients with severe respiratory failure on mechanical ventilation

	Unmatched groups		Propensity score-matched groups			
	Certified (*n* = 30,588)	Non-certified (*n* = 36,317)	Certified (*n* = 26,673)	Non-certified (*n* = 26,673)	Odds ratio (95% CI) or difference in days (95% CI)	*P* value
In-hospital mortality, *n* (%)	9404 (31)	13,668 (38)	8317 (31)	10,083 (38)	0.75 (0.72 to 0.77)	< 0.001
Reintubation^a^, *n* (%)	3476 (11)	4025 (11)	3025 (11)	2922 (11)	1.04 (0.99 to 1.10)	0.156
MV dependency^b^, *n* (%)	4227 (20)	5791 (26)	3790 (21)	4090 (25)	0.80 (0.76 to 0.84)	< 0.001
MV duration, day	9.0 (6.0–17)	10 (6.0–20)	9.0 (6.0–18)	10 (6.0–19)	− 2.77 (− 3.33 to − 2.21)	< 0.001
CCU length of stay^c^, day	10 (6.0–14)	8.0 (5.0–13)	9.0 (6.0–14)	9.0 (5.0–13)	0.55 (0.45 to 0.66)	< 0.001
ICU length of stay^c^, day	9.0 (5.0–13)	8.0 (5.0–13)	9.0 (5.0–13)	8.0 (5.0–13)	0.28 (0.16 to 0.40)	< 0.001
HCU length of stay^c^, day	5.0 (3.0–10)	7.0 (4.0–12)	5.0 (3.0–10)	8.0 (4.0–13)	− 1.63 (− 1.82 to − 1.44)	< 0.001
Hospital length of stay, day	24 (14–41)	26 (14–47)	24 (14–41)	25 (14–47)	− 5.54 (− 6.3 to − 4.79)	< 0.001

Data are presented as n (%) or median (interquartile range)

*MV* mechanical ventilation, *CCU* critical care unit, *ICU* intensive care unit, *HCU* high-care unit

^a^Within 7 days after the first extubation

^b^Using invasive mechanical ventilation at the time of hospital discharge for survivors

^c^Only patients who used these units

### Sensitivity and subgroup analyses

To avoid sample size reduction due to the propensity score-matched analysis and estimate the average treatment effect, we performed two sensitivity analyses using the stabilized inverse probability of treatment weighting method [28] with propensity score and logistic regression analysis for in-hospital mortality in the original cohort. In the logistic regression model, we included the same variables used in the propensity score calculation. The results were presented as median differences or odds ratios with 95% confidence intervals (CIs).

We also performed subgroup analyses according to age (≥ 80, 79–70, 69–60, or ≤ 59 years), use of vasopressor on the first day of MV, and admission to critical care units. We assumed that the impact of intensivists on patient care varies according to patient age, severity of illness, and treatment location, therefore, selecting these subgroups. These subgroup analyses were performed in the original cohort using the logistic regression model for in-hospital mortality. We also tested for an interaction between each subgroup and the certified group. Finally, we analyzed the interaction between each subgroup and the certified group. The interaction term for age was modeled with age as a continuous variable.

## Results

**Fig. 1:**
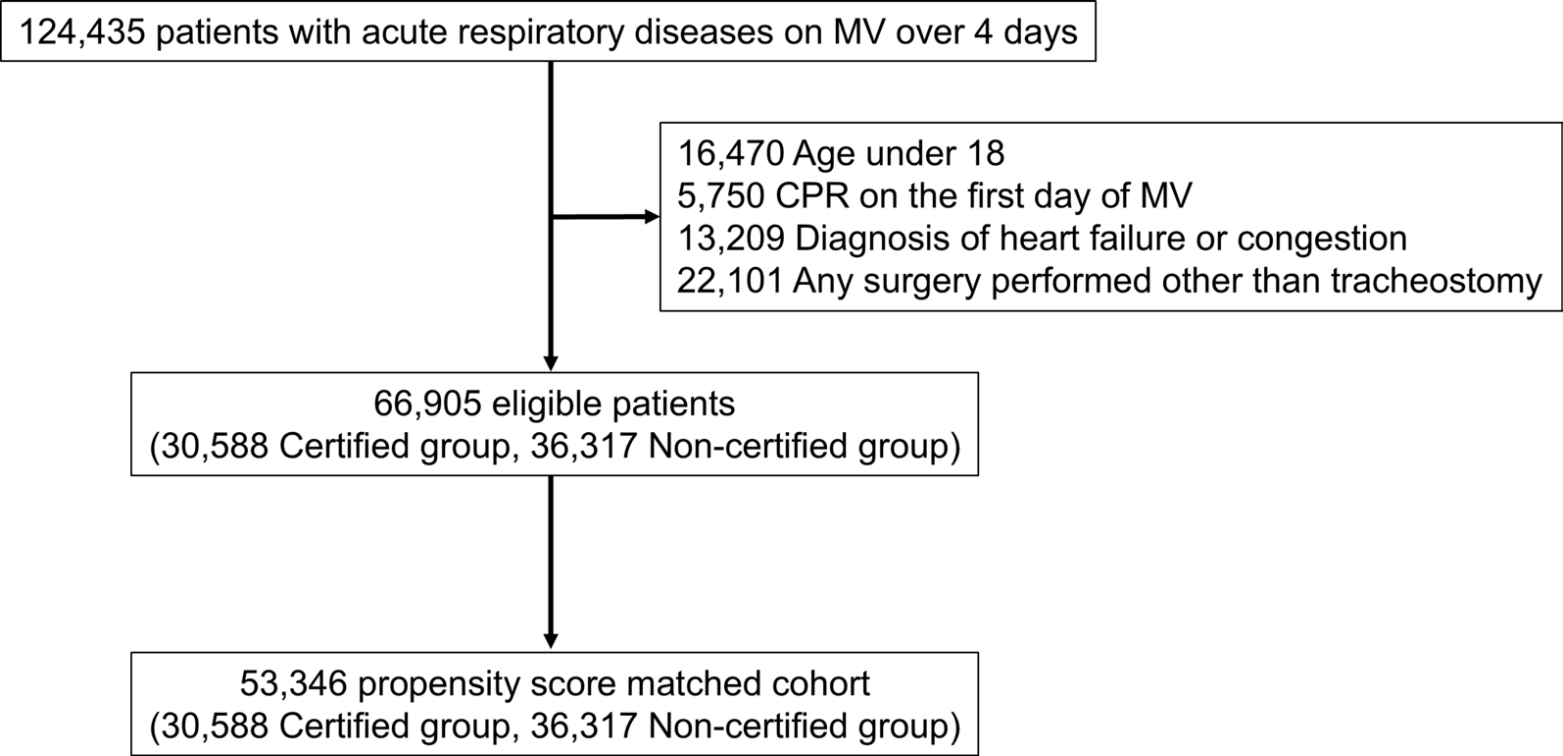
Flowchart of the patient selection. *MV* mechanical ventilation, *CPR* cardiopulmonary resuscitation

We identified 124,435 patients diagnosed with acute respiratory diseases on MV for 4 consecutive days within the first 7 days of admission during the study period. Of them, 66,905 were included in this study (Fig. 1). Using propensity score matching, we created a final study cohort with 26,673 matched pairs. The *c*-index of the propensity score was 0.66 (95% CI 0.65–0.66). The distributions of propensity scores before and after are shown in Figure S1.

Table 1 and Table S2 show baseline characteristics of the unmatched and propensity score-matched groups. In the original cohort, the certified group was ~ 4 years younger and had higher rates of ambulance use and vasopressor administration on the first day of MV than the non-certified group (certified group, 36%; non-certified group, 24%). After matching, patient characteristics were well balanced, with absolute standardized differences < 0.1 for all variables between the two groups.

Interventions after MV initiation are shown in Table 2. The use of dopamine, sivelestat, and high-dose methylprednisolone was less frequent in the certified group than that in the non-certified group (certified group vs. non-certified group: dopamine, 9.0% vs. 15%; sivelestat, 4.1% vs. 7.0%; high-dose methylprednisolone, 13% vs. 15%). The use of morphine and low-dose methylprednisolone was similar between certified and non-certified groups. All other interventions were used more frequently in the certified group than those in the non-certified group. Notably, a particularly large difference in ICU utilization was observed between the certified group and the non-certified group, both in absolute and relative terms (74% vs. 30%). These trends in usage differences between the groups were consistent before and after matching. Moreover, tracheostomy was performed ~ 3 days earlier in the certified group than in the non-certified group.

Clinical outcomes after 1:1 propensity score matching are shown in Table 3. After matching, the certified group had lower in-hospital mortality (odds ratio: 0.75; 95% confidence interval: 0.72–0.77) and MV dependency among survivors (odds ratio: 0.80; 95% confidence interval: 0.76–0.84) than the non-certified group. Additionally, the certified group had longer critical care unit stays but a shorter MV duration and hospital stays than the non-certified group.

The results of the sensitivity analyses using the stabilized inverse probability of treatment weighted by propensity score and the multivariable logistic regression model (detailed in Tables S3, S4, and S5) were consistent with those of the main analysis (IPTW analysis, odds ratio: 0.76; 95% confidence interval: 0.73–0.78; multivariable logistic regression analysis, odds ratio: 0.73; 95% confidence interval: 0.70–0.75).

**Fig. 2:**
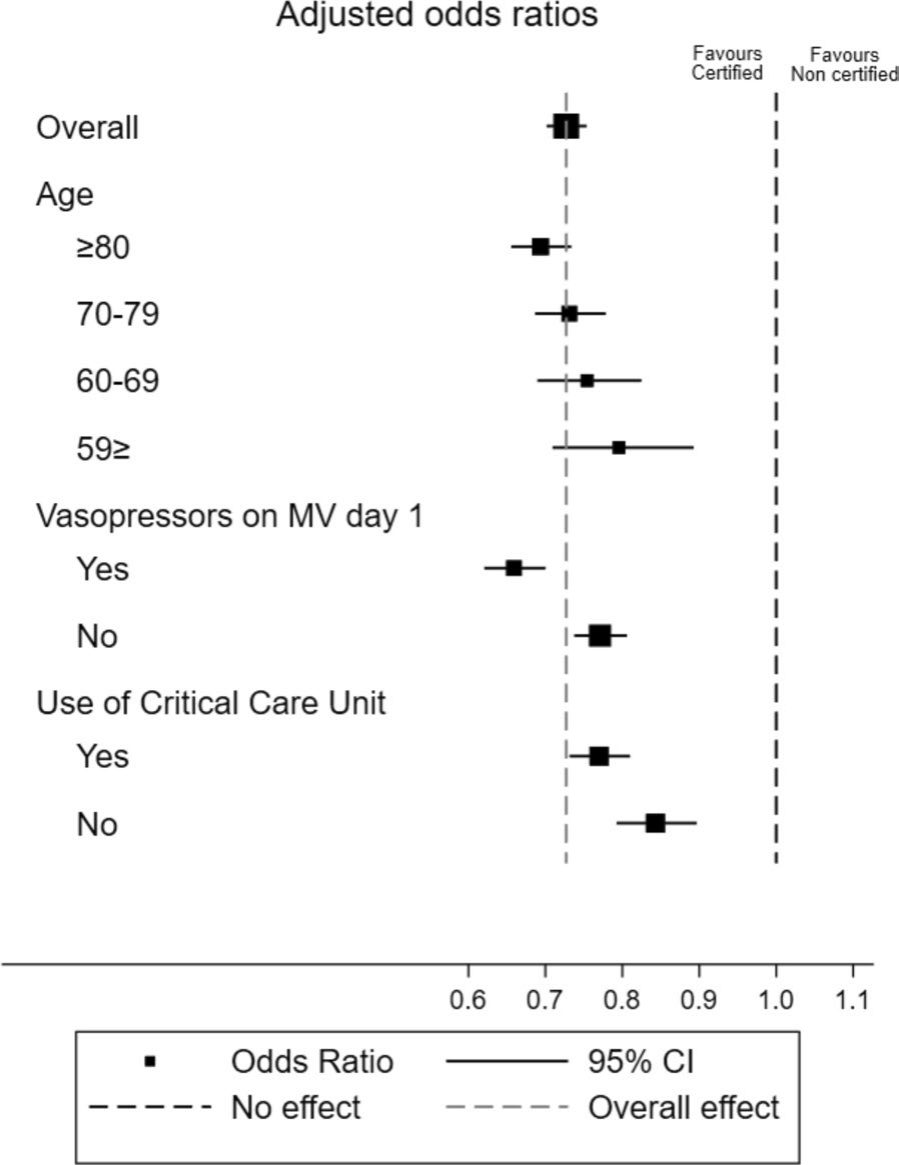
Forrest plots for subgroup analyses. *P* for interaction: age, *P* < 0.001; vasopressors on MV day 1, *P* < 0.001; use of critical care unit, *P* = 0.037. *MV* mechanical ventilation

Figure 2 shows the exploratory subgroup analysis for in-hospital mortality, highlighting that the greater benefits in the certified group were for older patients, those on vasopressors on the first day of MV, and those treated in critical care units. The *P*-values for the interaction terms for the effect of the certified group in each subgroup were as follows: age, *P* < 0.001; vasopressors on the first day of MV, *P* < 0.001; and use of critical care units, *P* = 0.037.

## Discussion

This nationwide cohort study of patients with severe respiratory failure found that admission at hospitals with board-certified intensive care training facilities was associated with lower hospital mortality than that at hospitals without board-certified facilities. In addition, the benefits of board-certified facilities against in-hospital mortality were more pronounced in older patients, those who required vasopressors on the first day of MV, and patients treated in critical care units. Regarding adjunctive treatments for severe respiratory failure, although most treatments were more common in hospitals with board-certified facilities, the use of dopamine, sivelestat, high-dose methylprednisolone, and parenteral nutrition were less frequent than that in hospitals without board-certified facilities.

Although the effect of intensivist staffing on patient outcomes is not fully understood yet [7, 11, 12], a systematic review in 2013 reported that ICU patients under high-intensity staffing models exhibited lower mortality compared with those under low-intensity staffing [13]. Our study showed the beneficial association between in-hospital mortality and board-certified intensive care training facilities that have at least one board-certified intensivist on staff. As of 2019, only 25% of all board-certified intensive care training facilities in Japan were reported to have a closed-model ICU [29]. In other words, overall, board-certified intensive care training facilities in Japan did not have a high-intensity intensivist staffing model. These findings suggest that factors influencing the prognosis of critically ill patients extend beyond the involvement of intensivists alone. For instance, other contributing factors may include the impact of a multidisciplinary team comprising ICU nurses, pharmacists, and clinical engineers as well as the hospital's available equipment, which may collectively be associated with improved patient outcomes. It is possible that board-certified facilities were more likely to already have these favorable factors before the board certification, which may have contributed to better patient prognoses and, as a result, acted as confounding factors. As this study is observational in nature, the possibility of residual confounding cannot be ruled out. Indeed, we were unable to adjust for the aforementioned factors or variables that directly reflect the severity of acute respiratory failure. Therefore, caution is warranted when interpreting these results, as the observed relationship between board-certified facilities and patient prognosis may represent an association rather than a causal effect.

Few studies have reported patient outcomes and treatment differences among different ICU staffing models. A prospective observational study of 1,075 lung injury patients across 23 US ICUs found lower in-hospital mortality in the closed model than that in the open model (adjusted odds ratio: 0.68; 95% CI 0.53–0.89); patients in the closed model used low tidal volume ventilation more frequently than those in the open model (difference of tidal volume: 1.4 mL/kg predicted body weight [95% CI 0.57–2.24]) [30, 31]. However, to the best of our knowledge, no study has evaluated the relationship between critical care expertise and the implementation of adjunctive therapies for the treatment of severe respiratory failure. In our study, board-certified facilities implemented numerous therapeutic interventions more frequently than non-certified facilities. However, dopamine, sivelestat, high-dose methylprednisolone, and parenteral nutrition were used less frequently in certified facilities. The lower frequency of parenteral nutrition in certified facilities likely resulted from a higher frequency of early enteral nutrition. These treatment choices in certified facilities are in line with currently available evidence in critical care settings [32–36]. Therefore, evidence-based treatment selection in board-certified facilities may contribute to lower in-hospital mortality. Our subgroup analyses, which indicated that ICU patients compared to non-ICU patients, and hypotensive patients with potentially more treatment options compared to non-hypotensive patients, may have benefited from accredited facilities, also supporting this view. However, an alternative perspective should be considered. In our study, there was a substantial difference in ICU utilization rates between board-certified and non-board-certified facilities. In Japan's acute care settings, decisions regarding treatment limitations are often made in the early stages of acute illness. At non-board-certified facilities, decisions to withhold advanced treatment in the early stages of acute illness may have been made more frequently, which may have, in turn, influenced choices of adjunctive therapies and potentially resulted in higher in-hospital mortality.

The shortage of intensivists has long been a problem [37, 38], and our study revealed that around 30% of MV patients are managed in general wards at board-certified facilities, whereas ~ 70% are managed in general wards at non-board-certified facilities. This finding may also account for the shorter CCU length of stay in non-board-certified facilities compared to board-certified facilities. Furthermore, with an aging population, the need for intensive care is expected to increase [39–41]. Our subgroup analysis suggested that as age increases, patients may be more likely to benefit from board-certified facilities. Although the causal relationship between certification itself and patient outcomes remains uncertain in our study, increasing the number of board-certified facilities that meet the same level of standards as set by JSICM may enhance treatment outcomes nationwide and help address the future healthcare needs of Japan’s aging society.

To the best of our knowledge, this study included a much larger population than existing studies, evaluating both the outcomes and treatments provided to patients with severe respiratory failure in board-certified facilities. Using a national administrative database, this study included over 60,000 patients with severe respiratory failure and elucidated differences in treatments and outcomes associated with critical care expertise. However, some limitations should be acknowledged. First, owing to the characteristics of the data used, information on the settings of MV, non-invasive ventilation, and prone positioning could not be obtained, leaving these clinical practices unclear. Second, we presented clinical practices during the period before the onset of the coronavirus disease 2019 pandemic, and current practices may have changed. Third, this study was conducted in Japan, and it is unclear whether our findings can be applied outside of Japan. Fourth, we included in the study patients with diagnoses for which bilateral lesions were considered possible by us, focusing on cases of severe respiratory failure suggestive of ARDS. This determination was based solely on clinical judgment. Therefore, some patients may have only unilateral lesions in our study. However, the target condition of our study is severe respiratory failure. We believe that patients who were given a diagnosis related to acute respiratory failure and required MV for more than 4 days could be clinically treated as having severe respiratory failure. Fifth, we conducted exploratory subgroup analyses. In interpreting these results, no adjustments for multiplicity were applied, which entails a risk of a type 1 error. Finally, owing to the observational nature of the study, there may remain unmeasured or unknown confounding factors. For example, this includes the specialized personnel and equipment that the hospital had in place before certification, as well as indicators that directly represent the severity of acute respiratory failure in patients. However, conducting randomized controlled trials in this area is challenging. Consequently, large-scale international observational studies that comprehensively gather information on confounders like severity are essential.

## Conclusions

We evaluated differences in adjunctive therapies and prognosis of patients with severe respiratory failure between board-certified intensive care training facilities and non-board-certified facilities. Board-certified intensive care training facilities implemented several different adjunctive treatments for severe respiratory failure compared to non-board-certified facilities, and board-certified facilities were associated with lower in-hospital mortality. While a correlation exists between the certification of intensive care training facilities in Japan and both treatment choices and patient outcomes, establishing a causal relationship remains uncertain. Further research is necessary to determine the most effective ways to enhance patient outcomes by certifying intensive care training facilities.

## Supplementary Information

Supplementary Material 1.

## Data Availability

The dataset(s) supporting the conclusions of this article are available from the corresponding author TM upon reasonable request.

## Abbreviations

ARDS: Acute respiratory distress syndrome
CIs: Confidence intervals
DPC: Diagnostic procedure combination
HCU: High-care unit
ICD-10: International Classification of Diseases, Tenth Revision
ICU: Intensive care unit
IQR: Interquartile range
JSICM: Japanese Society of Intensive Care Medicine
MV: Mechanical ventilation
