# Supplementary material for: Impact of board-certified intensive care training facilities on choice of adjunctive therapies and prognosis of severe respiratory failure: a nationwide cohort study
Source: J Intensive Care. 2024 Dec 19;12:52. doi: 10.1186/s40560-024-00766-8 (PMC11658443; doi:10.1186/s40560-024-00766-8)

**Table S1: ICD-10 codes.**

|  | ICD-10 codes |
| --- | --- |
| Diagnosis associated with acute respiratory disease ^a^ |  |
| Bacterial pneumonia | A01, A02, A24, A40, A41, A48, A49, A54, A69, A70, J13, J14, J15, J16, J20, J85 |
| Interstitial pneumonia | B22, J70, J84, M05, M32, M33, M35 |
| Acute respiratory distress syndrome | J80 |
| Allergic | J45, J67, J82 |
| Viral pneumonia | B01, B05, B06, B25, J11, J12, J20, J21, U07, U08, U09, U10 |
| Tuberculosis | A15, A16, A19, B90, J65 |
| Mycobacterium avium complex | A31, B20 |
| Aspergillus | B44 |
| Pneumocystis pneumonia | B20, B59 |
| Any pneumonia or bronchitis without specified causative microorganisms ^b^ | C34, J18, J20, J21, J40, J41, J42, J44, J68, J69, J70, J80, J95, J96, O29, O99, U04 |
| Heart failure or congestion | E05, I09, I11, I50, J81, J94, J98, O29 |

ICD-10: International Classiﬁcation of Diseases, Tenth Revision.

^a^ Diagnosis recorded in “main diagnosis,” “admission-precipitating diagnosis,” “most resource-consuming diagnosis,” or “second resource-consuming diagnosis”. Some patients were assigned multiple associated diagnosis over several categories.

^b^ Any pneumonia or bronchitis without specified causative microorganisms.

**Table S2: Baseline characteristic of patients with severe respiratory failure on mechanical ventilation.**

|  | Unmatched groups | | | Propensity score-matched groups | | |
| --- | --- | --- | --- | --- | --- | --- |
|  | Certified  (n =30,588) | Non-certified (n = 36,317) | ASD | Certified  (n =26,673) | Non-certified  (n = 26,673) | ASD |
| Myocardial infarction, n (%) | 767 (2.5) | 1,002 (2.8) | 0.016 | 697 (2.6) | 706 (2.6) | 0.002 |
| Congestive heart failure, n (%) | 4,925 (16) | 8,571 (24) | 0.189 | 4,745 (18) | 4,828 (18) | 0.008 |
| Peripheral vascular disease, n (%) | 453 (1.5) | 517 (1.4) | 0.005 | 399 (1.5) | 403 (1.5) | 0.001 |
| Cerebrovascular disease, n (%) | 2,648 (8.7) | 3,702 (10) | 0.053 | 2,462 (9.2) | 2,462 (9.2) | <0.001 |
| Dementia, n (%) | 1,142 (3.7) | 2,447 (6.7) | 0.135 | 1,139 (4.3) | 1,136 (4.3) | 0.001 |
| Chronic pulmonary disease, n (%) | 5,740 (19) | 9,714 (27) | 0.191 | 5,530 (21) | 5,641 (21) | 0.010 |
| Rheumatologic disease, n (%) | 1,133 (3.7) | 995 (2.7) | 0.055 | 912 (3.4) | 858 (3.2) | 0.011 |
| Peptic ulcer disease, n (%) | 965 (3.2) | 840 (2.3) | 0.052 | 749 (2.8) | 733 (2.7) | 0.004 |
| Mild liver disease, n (%) | 761 (2.5) | 838 (2.3) | 0.012 | 671 (2.5) | 643 (2.4) | 0.007 |
| Diabetes without chronic complications, n (%) | 4,112 (13) | 5,457 (15) | 0.045 | 3,763 (14) | 3,836 (14) | 0.008 |
| Diabetes with chronic complications, n (%) | 840 (2.7) | 1,067 (2.9) | 0.012 | 749 (2.8) | 775 (2.9) | 0.006 |
| Hemiplegia or paraplegia, n (%) | 159 (0.5) | 188 (0.5) | 0 | 143 (0.5) | 149 (0.6) | 0.003 |
| Renal disease, n (%) | 1,568 (5.1) | 1,744 (4.8) | 0.015 | 1,359 (5.1) | 1,354 (5.1) | 0.001 |
| Any malignancy, including lymphoma and leukemia, except malignant neoplasm of skin n (%) | 2,954 (9.7) | 3,145 (8.7) | 0.035 | 2,510 (9.4) | 2,539 (9.5) | 0.004 |
| Moderate or severe liver disease, n (%) | 205 (0.7) | 113 (0.3) | 0.051 | 129 (0.5) | 99 (0.4) | 0.017 |
| Metastatic solid cancer, n (%) | 462 (1.5) | 543 (1.5) | 0.001 | 422 (1.6) | 439 (1.6) | 0.005 |
| AIDS/HIV, n (%) | 42 (0.1) | 12 (0.0) | 0.036 | 17 (0.1) | 12 (0.0) | 0.008 |

AIDS, acquired Immunodeficiency Syndrome; HIV, human immunodeficiency virus; ASD, absolute standardized difference.

**Table S3:** **Multivariable logistic regression analysis for hospital mortality.**

|  | Adjusted odds ratio (95% confidence interval) | *P* value |
| --- | --- | --- |
| **Board-certified facility** | **0.73 (0.70 to 0.75)** | **<0.001** |
| Age, years | 1.04 (1.03 to 1.04) | <0.001 |
| Male | 1.21 (1.16 to 1.26) | <0.001 |
| Body mass index, kg/m2 | 0.98 (0.98 to 0.98) | <0.001 |
| Emergency admission | 1.25 (1.15 to 1.37) | <0.001 |
| Ambulance use | 1.19 (1.14 to 1.24) | <0.001 |
| Smoking | 0.93 (0.89 to 0.97) | 0.001 |
| Myocardial infarction | 1.16 (1.04 to 1.28) | 0.005 |
| Congestive heart failure | 0.91 (0.87 to 0.95) | <0.001 |
| Peripheral vascular disease | 0.94 (0.82 to 1.08) | 0.41 |
| Cerebrovascular disease | 1.28 (1.21 to 1.35) | <0.001 |
| Dementia | 0.86 (0.80 to 0.93) | <0.001 |
| Chronic pulmonary disease | 0.64 (0.61 to 0.67) | <0.001 |
| Rheumatologic disease | 1.19 (1.08 to 1.31) | <0.001 |
| Peptic ulcer disease | 0.72 (0.64 to 0.80) | <0.001 |
| Mild liver disease | 1.39 (1.25 to 1.55) | <0.001 |
| Diabetes without chronic complications | 0.78 (0.75 to 0.82) | <0.001 |
| Diabetes with chronic complications | 0.80 (0.72 to 0.89) | <0.001 |
| Hemiplegia or paraplegia | 0.53 (0.39 to 0.74) | <0.001 |
| Renal disease | 1.65 (1.53 to 1.78) | <0.001 |
| Any malignancy, including lymphoma and leukemia, except malignant neoplasm of skin | 1.80 (1.70 to 1.90) | <0.001 |
| Moderate or severe liver disease | 2.94 (2.32 to 3.71) | <0.001 |
| Metastatic solid cancer | 3.38 (2.95 to 3.87) | <0.001 |
| AIDS/HIV | 1.18 (0.63 to 2.20) | 0.606 |
| Any pneumonia or bronchitis | 0.95 (0.91 to 0.99) | 0.02 |
| Bacterial pneumonia | 1.13 (1.08 to 1.18) | <0.001 |
| Viral pneumonia | 1.08 (1.00 to 1.16) | 0.045 |
| Interstitial pneumonia | 2.63 (2.50 to 2.77) | <0.001 |
| Allergic | 0.69 (0.63 to 0.76) | <0.001 |
| ARDS | 1.67 (1.57 to 1.78) | <0.001 |
| Tuberculosis | 0.80 (0.71 to 0.90) | <0.001 |
| Mycobacterium avium complex | 1.49 (1.28 to 1.74) | <0.001 |
| Aspergillus | 1.77 (1.51 to 2.08) | <0.001 |
| Pneumocystis pneumonia | 1.72 (1.43 to 2.07) | <0.001 |
| Vasopressors on MV day 1 | 1.45 (1.40 to 1.51) | <0.001 |
| ECMO on MV day 1 | 1.06 (0.42 to 2.69) | 0.904 |

AIDS, acquired Immunodeficiency Syndrome; HIV, human immunodeficiency virus; ARDS, acute respiratory distress syndrome; ECMO, extracorporeal membrane oxygenation; MV, mechanical ventilation.

**Table S4.** **Absolute standardized differences of covariate for IPTW analysis.**

|  | ASD |
| --- | --- |
| Age | 0.001 |
| Male | 0.001 |
| Body mass index | 0.012 |
| Emergency admission | <0.001 |
| Ambulance use | 0.001 |
| Smoking | 0.002 |
| Bacterial pneumonia | 0.003 |
| Interstitial pneumonia | 0.001 |
| ARDS | 0.002 |
| Allergic | 0.002 |
| Viral pneumonia | <0.001 |
| Tuberculosis | 0.001 |
| Mycobacterium avium complex | <0.001 |
| Aspergillus | <0.001 |
| Pneumocystis pneumonia | 0.001 |
| Any pneumonia or bronchitis | 0.002 |
| Vasopressors on MV day 1 | 0.001 |
| ECMO on MV day 1 | 0.026 |
| Myocardial infarction | <0.001 |
| Congestive heart failure | 0.001 |
| Peripheral vascular disease | <0.001 |
| Cerebrovascular disease | <0.001 |
| Dementia | 0.001 |
| Chronic pulmonary disease | 0.005 |
| Rheumatologic disease | <0.001 |
| Peptic ulcer disease | 0.001 |
| Mild liver disease | 0.001 |
| Diabetes without chronic complications | <0.001 |
| Diabetes with chronic complications | <0.001 |
| Hemiplegia or paraplegia | <0.001 |
| Renal disease | 0.001 |
| Any malignancy, including lymphoma and leukemia, except malignant neoplasm of skin | 0.001 |
| Moderate or severe liver disease | 0.001 |
| Metastatic solid cancer | 0.001 |
| AIDS/HIV | 0.001 |

ARDS, acute respiratory distress syndrome; MV, mechanical ventilation; ECMO, extracorporeal membrane oxygenation; AIDS, acquired Immunodeficiency Syndrome; HIV, human immunodeficiency virus.

**Table S5: Result of inverse probability of treatment weighting analysis for hospital mortality.**

|  | Adjusted odds ratio (95% confidence interval) | *P* value |
| --- | --- | --- |
| Board-certified facility | 0.76 (0.73 to 0.78) | <0.001 |

**Figure S1: Distributional balance for Propensity score before and after matching**


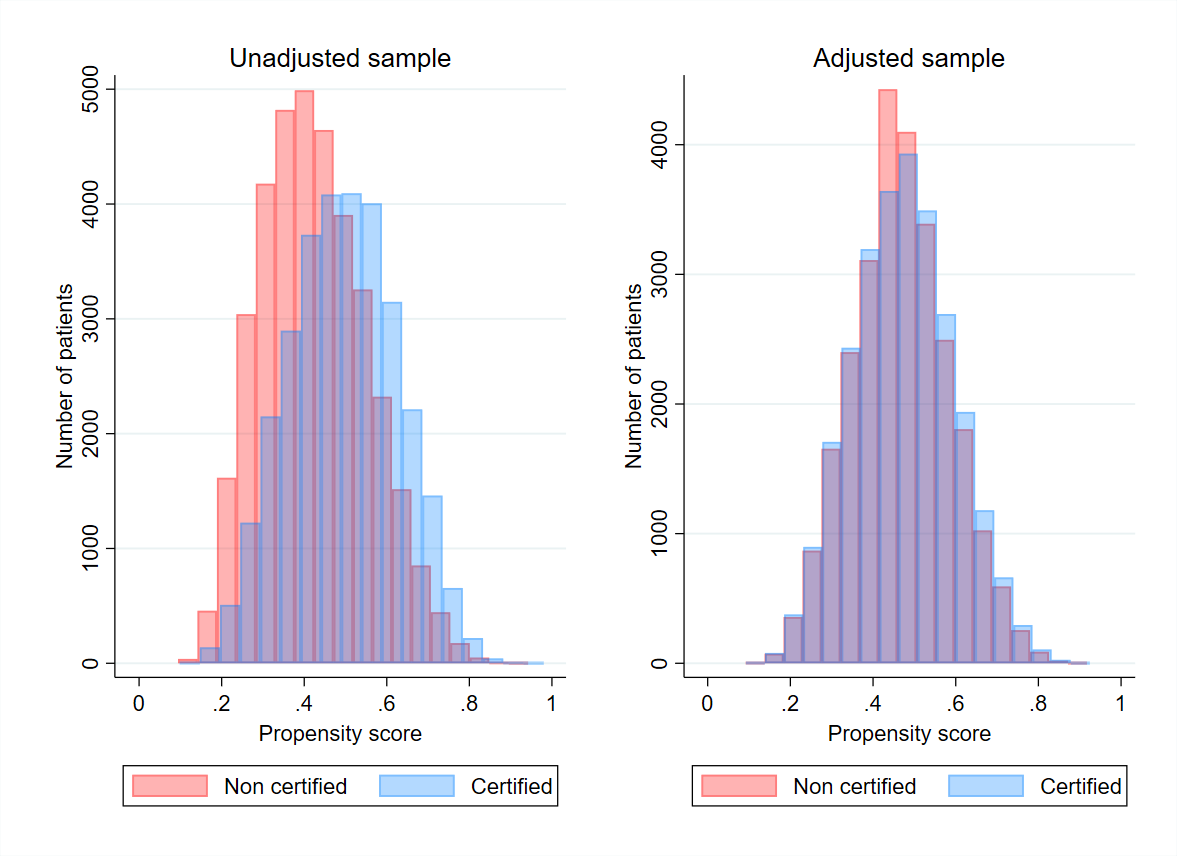

Supplement: Supplementary file 1 — Supplementary Material 1. [file 40560_2024_766_MOESM1_ESM.docx]
